# Duplication of the *NPHP1* gene in patients with autism spectrum disorder and normal intellectual ability: a case series

**DOI:** 10.1186/s12991-014-0022-2

**Published:** 2014-08-06

**Authors:** Yuka Yasuda, Ryota Hashimoto, Ryoko Fukai, Nobuhiko Okamoto, Yoko Hiraki, Hidenaga Yamamori, Michiko Fujimoto, Kazutaka Ohi, Masako Taniike, Ikuko Mohri, Mitsuko Nakashima, Yoshinori Tsurusaki, Hirotomo Saitsu, Naomichi Matsumoto, Noriko Miyake, Masatoshi Takeda

**Affiliations:** 1 Department of Psychiatry, Osaka University Graduate School of Medicine, D3, 2-2, Yamadaoka, Suita 565-0871, Osaka, Japan; 2 Molecular Research Center for Children’s Mental Development, United Graduate School of Child Development, Osaka University, D3, 2-2, Yamadaoka, Suita 565-0871, Osaka, Japan; 3 Department of Human Genetics, Yokohama City University Graduate School of Medicine, 3-9 Fukuura, Kanazawa-ku 236-0004, Yokohama, Japan; 4 Department of Neurology and Stroke Medicine, Yokohama City University Graduate School of Medicine, 3-9 Fukuura, Kanazawa-ku 236-0004, Yokohama, Japan; 5 Division of Medical Genetics, Osaka Medical Center and Research Institute for Maternal and Child Health, Izumi 594-1101, Japan; 6 Hiroshima Municipal Center for Child Health and Development, Hiroshima 732-0052, Japan; 7 Department of Molecular Neuropsychiatry, Osaka University Graduate School of Medicine, D3, 2-2, Yamadaoka, Suita 565-0871, Osaka, Japan

**Keywords:** Autism spectrum disorder, Copy-number variants, Duplication, Nephronophthisis 1 gene, Intelligence

## Abstract

Autism spectrum disorder is a neurodevelopmental disorder characterized by impairments in social interactions, reduced verbal communication abilities, stereotyped repetitive behaviors, and restricted interests. It is a complex condition caused by genetic and environmental factors; the high heritability of this disorder supports the presence of a significant genetic contribution. Many studies have suggested that copy-number variants contribute to the etiology of autism spectrum disorder. Recently, copy-number variants of the *nephronophthisis 1* gene have been reported in patients with autism spectrum disorder. To the best of our knowledge, only six autism spectrum disorder cases with duplications of the *nephronophthisis 1* gene have been reported. These patients exhibited intellectual dysfunction, including verbal dysfunction in one patient, below-average verbal intellectual ability in one patient, and intellectual disability in four patients.

In this study, we identified *nephronophthisis 1* duplications in two unrelated Japanese patients with autism spectrum disorder using a high-resolution single-nucleotide polymorphism array. This report is the first to describe a *nephronophthisis 1* duplication in an autism spectrum disorder patient with an average verbal intelligence quotient and an average performance intelligence quotient. However, the second autism spectrum disorder patient with a *nephronophthisis 1* duplication had a below-average performance intelligence quotient. Neither patient exhibited physical dysfunction, motor developmental delay, or neurological abnormalities. This study supports the clinical observation of *nephronophthisis 1* duplication in autism spectrum disorder cases and might contribute to our understanding of the clinical phenotype that arises from this duplication.

## Background

Autism spectrum disorder (ASD) is a neurodevelopmental condition characterized by compromised social interactions, reduced verbal communication abilities, stereotyped repetitive behaviors, and restricted interests [1]. The prevalence of ASD is estimated to be 62 in 10,000 individuals, and the number of diagnosed cases has increased in the past decade [2],[3]. The etiologies of ASD are complex and unclear, and both genetic and environmental factors are hypothesized to be contributors. The following genetic factors have been reported to be associated with of ASD: 2,135 non-syndromic autism-related genes, 99 syndromic autism-related genes, 4,544 copy-number variations (CNVs), and 158 linked regions [4]. Copy-number alterations in the *nephronophthisis 1* gene (*NPHP1*; NM_000272.3) which is located in the 2q13 region have been associated with ASD [5]-[9]. The genomic region that surrounds *NPHP1* is flanked by two inverted low-copy repeats (LCRs), which include 45-kb direct repeats; therefore, deletion or duplication can easily occur in this region via non-allelic homologous recombination, which results in *NPHP1* copy-number changes [10]. In humans, homozygous or compound heterozygous deletions and loss-of-function mutations in *NPHP1* cause ciliopathies such as juvenile nephronophthisis (MIM# 256100), Joubert syndrome type 4 (MIM# 609583), and Senior-Loken syndrome 1 (MIM# 266900) [10],[11]. In a 2007 study, 1 of 14 patients with Joubert syndrome type 4 exhibited ASD [12]. Additional studies have confirmed the presence of *NPHP1* deletions in individuals with ASD [5]-[8]. Duplication of the *NPHP1* gene has been identified in ASD patients and controls [12],[13]. Whether the CNV in this gene is a normal variant is unknown [7],[13]. Six individuals with ASD have been reported to have *NPHP1* duplications confirmed by a validation analysis [7],[13],[14] (Table 1). The *NPHP1* gene and its product are associated with the primary cilium and basal body (PC/BB) and subcellular organelles [15]. The PC/BB control diverse cellular processes such as cell division, differentiation, migration, and planar cell polarity through signaling pathways [11],[15]. These molecular findings and reports of the clinical phenotypes in patients with an altered *NPHP1* gene support the hypothesis that the gene is associated with brain development and cognitive impairment. However, no functional study has reported a correlation between the *NPHP1* gene and intelligence.

**Table 1:** **Characteristics of individuals with a duplication of** ***NPHP1*** **gene in our study and previous studies**

**Author,year**	**ID in the study**	**Diagnosis**	**Age(years)**	**Gender**	**Cognitive profile**	**Dysmorphic features**	**Clinical profile**
Yasuda Y. et al. 2014	Patient 1	ASD	24	Male	FIQ 110	No dysmorphic features were observed	No evidence of epilepsy was observed. The neurological examination was normal. The function of visceral organs, including the kidneys, was normal
					PIQ 98		
					VIQ 117		
	Patient 2	ASD	33	Male	FIQ 80	No dysmorphic features were observed	No evidence of epilepsy was observed. The neurological examination was normal. The function of visceral organs, including the kidneys, was normal
					VIQ 86		
					PIQ 76		
Kaminsky EB et al. 2011 [14]	ISCA00000020	ASD	NA	NA	Intellectual disability was detected	NA	Developmental delay was observed
	ISCA00000128	ASD	NA	NA	Intellectual disability was detected.	NA	Developmental delay was observed
	ISCA00000949	ASD	NA	NA	Intellectual disability was detected	NA	Developmental delay was observed
	ISCA00001175	ASD	NA	NA	Intellectual disability was detected	NA	Developmental delay was observed
Pinto D et al. 2010 [7]	5036_4	Autism	NA	Male	Below-average VIQ and average non-verbal IQ were detected	No dysmorphic features were observed	No evidence of epilepsy was observed
Baris H et al. 2006 [13]		PDD/ADHD/Obsessive compulsive disorder	12		Language-based learning disability was detected	Mid-face hypoplasia, bilateral epicanthal folds, long palpebral fissures, synophrys, upturned nose with a depressed nasal root and retrognathia, and relative macrocephaly were observed	Speech delay and abnormal skin lesions were observed. The neurological examination was normal. Brain MRI was normal. A renal ultrasound scan was normal

*ASD* autism spectrum disorder, *PDD* pervasive developmental disorder, *ADHD* attention deficit hyperactivity disorder, *NA* not available, *FIQ* full-scale IQ, *PIQ* performance IQ, *VIQ* verbal IQ.

Of the six individuals who have been identified with ASD and *NPHP1* duplications, one patient had a verbal intelligence quotient (VIQ) that was below average [7], one patient exhibited verbal dysfunction [13], and four other patients had intellectual disabilities [14]. We identified an *NPHP1* duplication in two unrelated Japanese patients with ASD using a high-resolution single-nucleotide polymorphism array. One of these patients had a normal VIQ and performance IQ (PIQ). The other ASD patient had a normal VIQ, with a PIQ that was below average. As previously described, *NPHP1* duplications have been observed in a number of ASD cohorts. No reports have delineated the clinical features of patients who harbor an *NPHP1* duplication. Clinical data from patients with ASD will facilitate an understanding of the contribution of this CNV to the development of the disorder. To the best of our knowledge, this report is the first to describe ASD cases with *NPHP1* duplications but without dysmorphic features or intellectual dysfunction.

## Case presentation

### Case 1

Patient 1 was a 24-year-old male with ASD. He was born vaginally at 37 weeks and had a birth weight of 2,190 g (−1.43 standard deviation, SD); his mother had pregnancy-induced hypertension. The family history was unremarkable. The patient began to walk independently at 15 months of age. During his childhood, he had difficulty with social communication and exhibited distress during the first grade from changes in the elementary school environment. He was first seen in the clinic when he was in the fifth grade because of the development of a tic, which improved without treatment. When the patient was a college senior, his symptoms worsened, and he was first diagnosed with ASD at 24 years of age by a child psychiatrist. At 26 years of age, his intellectual ability was within the normal range (a full-scale IQ (FIQ) of 110, a VIQ of 117, and a PIQ of 98). He was not dysmorphic. The neurological examination and the functioning of visceral organs, including the kidneys, were normal. Brain magnetic resonance imaging (MRI) at 24 years of age was reported as normal. His parents were phenotypically normal.

### Case 2

Patient 2 was a 33-year-old male with ASD. He was delivered by Cesarean section, and neonatal asphyxia was caused by dystocia following an uneventful pregnancy. His birth weight was 4,070 g (+2.6 SD). The patient developed tics in elementary school and had difficulty with social communication. He was frequently a victim of bullying and exhibited distress over changes in his environment. At 17 years of age, he was diagnosed with schizophrenia, which resulted in repeated hospitalizations. When he was 33 years old, a new doctor noticed his communication impairments and stereotyped behaviors. The doctor initially referred him to a child psychiatrist, and his diagnosis was changed to ASD and schizophrenia.

At 36 years of age, his intellectual ability was within the normal range (FIQ, 80; VIQ, 86); his PIQ was below average (PIQ, 76). He had no dysmorphic features. The neurological examination and the functioning of visceral organs, including the kidneys, were normal. Brain MRI at 33 years of age was reported as normal. His father was phenotypically normal, whereas his mother was affected with schizotypal personality disorder.

### Assessment of ASD

The patients were diagnosed by at least two trained child psychiatrists and/or child neurologists according to the criteria of the Diagnostic and Statistical Manual of Mental Disorders, 5th Edition (DSM-5). The assessments were performed using unstructured or semi-structured behavioral observation and interviews with the patients and their parents or caregivers [16]. Additionally, the participants met the ASD criteria of the Autism Diagnostic Interview-Revised (ADI-R) [17]. The Pervasive Developmental Disorders Autism Society Japan Rating Scale (PARS) [18] and the Japanese version of the Asperger’s Questionnaire (AQ-J) [19] were used to evaluate ASD-specific behaviors and symptoms. The patients were recruited at Osaka University Hospital.

### Cognitive tests

The Japanese version of the full-scale Wechsler Adult Intelligence Scale-III (WAIS-III) [20] was used to obtain the IQ data.

### Copy-number analysis

Genomic DNA was extracted from whole blood using the QIAamp DNA Blood Maxi Kit (Qiagen, Hilden, Germany). The biological parentage was confirmed using nine polymorphic microsatellite markers (*D1S450*, *D1S206*, *D5S630*, *D5S410*, *D9S285*, *D9S1776*, *D13S217*, *D14S276*, and *D14S985*). The amplicons were separated on a Genetic Analyzer 3500 (Life Technologies, Inc., Carlsbad, CA, USA) and were analyzed using GeneMapper software, version 4.1 (Life Technologies, Inc.).

CNVs were analyzed using the CytoScan™ HD array (Affymetrix, Santa Clara, CA, USA) and Chromosome Analysis Suite (ChAS) software, version 1.2 (Affymetrix) according to the manufacturer’s instructions. The detection conditions in ChAS were set as follows: a confidence value of 90%, 20 contiguous probes, and a size larger than 100 kb for duplications; and a confidence value of 89%, 20 contiguous markers, and a size larger than 10 kb for deletions. The copy-number alterations detected by the microarray analysis were validated by quantitative real-time polymerase chain reaction (PCR) using Rotor-Gene Q (Qiagen). *STXBP1* and *FBN1* genes were used as reference genes. The PCR conditions and primer sequences are available upon request. Quantitative PCR was conducted in duplicate.

Using a high-resolution microarray analysis, we found a 110-862-kb duplication that involved the *NPHP1* gene in these two patients (minimum, chr2 110, 873, 834-110, 983, 413 bp and maximum, chr2 110, 504, 317-111, 365, 995 bp based on the University of California Santa Cruz Genome Browser, build 37 [hg19]). The entire *NPHP1* gene and the partial *MALL* gene are protein-coding genes that are located between two LCRs (see Additional file 1: Figure S1). We confirmed the presence of these *de novo* duplications in both patients using quantitative real-time PCR with DNA (see Additional file 2: Figure S2). Additionally, the biological parentage was confirmed.

## Conclusions

We examined two Japanese ASD cases with *NPHP1* duplications and delineated their clinical features. Previous studies have described ASD cases with *NPHP1* duplications; however, these cases were associated with FIQ or VIQ impairment [7],[13],[14]. By contrast, we found that one patient with ASD in this study had an average intellectual ability. There is a possibility that the difference in cognitive ability could be caused by other genetic abnormalities and not by the *NPHP1* gene. Details of microarray-based genomic profiling in the region of the duplication of the *NPHP1* gene are shown in our two cases and six cases reported in previous studies (Additional file 3: Table S1). No specific gene, which is able to distinguish between our case with normal range of intellectual ability and cases in our study and previous studies with intellectual impairment, was found. We found a paternal CNV in our two cases in different region, where the genes did not exist, suggesting that no other gene might cause functional impact on this cognitive phenotype. On the other hand, the information of other CNVs present in six cases with intellectual impairment in previous studies was not available. It is possible that unknown CNVs other than duplication of the *NPHP1* gene might be responsible for intellectual impairment in previous six cases.

One of the six previously described ASD cases had dysmorphic features [13]. An additional ASD case did not exhibit dysmorphic features [7], and data regarding dysmorphic features were not available for the other ASD cases [14] (Table 1). Our cases did not exhibit dysmorphic features. These findings suggest that patients with ASD and an *NPHP1* duplication might not exhibit dysmorphic features. Previously described cases with *NPHP1* duplications that were not associated with ASD demonstrated various phenotypes (Table 1) [13],[21]. It is unclear whether an *NPHP1* duplication is the causative factor of ASD.

The patients in this study had ASD features from early childhood; however, they were not diagnosed with ASD until adulthood. This delayed diagnosis might have resulted from the patients having an average FIQ. Individuals with ASD vary in intelligence, which ranges from having an intellectual disability to a high IQ [22]. Individuals with a normal or high IQ are frequently difficult to diagnose as having ASD. In addition, the diagnosis of ASD might not have been well understood during their childhood years, which could have resulted in a delayed diagnosis. Patient 2 suffered from schizophrenia and secondary ASD; a high rate of comorbidity with other psychiatric disorders has been observed in adults with ASD [23]. However, it is not possible to confirm whether this patient had two independent disorders. A duplication of the *NPHP1* gene occurred in both cases in this study; however, this duplication has been observed in controls. To clarify whether the *NPHP1* gene is a disease-causing gene or a disease-associated factor, further investigation of this locus in larger samples is warranted.

## Consent

A description of the study was provided to each subject, and written informed consent was obtained from each subject regarding publication of this case report and any accompanying data. Peripheral blood samples from the patients and their families (when available) were collected after obtaining informed consent. This study was performed according to the Declaration of Helsinki of the World Medical Association; it was approved by the ethics committee at Osaka University and the institutional review board of Yokohama City University School of Medicine. Copies of the signed informed consent forms are available for review by the Editor-in-Chief of this journal.

## Abbreviations

ADI-R: Autism Diagnostic Interview-Revised

AQ-J: Japanese version of the Asperger’s Questionnaire

ASD: autism spectrum disorder

CNVs: copy-number variations

DSM-5: Diagnostic and Statistical Manual of Mental Disorders, 5th Edition

FIQ: full-scale intelligence quotient

*NPHP1*: *nephronophthisis 1*

PARS: Pervasive Developmental Disorders Autism Society Japan Rating Scale

PIQ: performance intelligence quotient

SD: standard deviation

VIQ: verbal intelligence quotient

WAIS-III: Wechsler Adult Intelligence Scale-III

## Competing interests

All authors declare that they have no competing interests.

## Authors’ contributions

NM, NM, and RH supervised the project and were critically involved in the design, analysis, interpretation, and coordination of the data. Additionally, they helped draft the manuscript. RF, RH, and YY wrote the draft of the manuscript. IM, MT, MT, NO, RH, YH, and YY were critically involved in the data collection and interpretation. HS, MN, NM, NM, RF, and YT performed the molecular genetic analyses and interpreted the data. All of the authors read and approved the final manuscript.

## Additional files

## Supplementary Material

Additional file 1: Figure S1. *De novo* 2p13 duplication in two individuals with ASD. Single-nucleotide polymorphism array profiles in the 2p13 region are shown for a control patient (top), patient 1 (second row), and patient 2 (third row). The location of *NPHP1*, other genes, and low-copy repeats (LCRs) (gray box) are depicted based on the University of California Santa Cruz Genome Browser, build 37 (hg19) (bottom). Click here for file

Additional file 2: Figure S2. Confirmation of the *de novo* 2p13 duplication in two individuals with ASD. Gene expression was determined by quantitative real-time PCR, and the relative concentration of *NPHP1* is shown on the *Y*-axis. Duplication was confirmed in the two patients (Pt 1 and Pt 2) with ASD. Click here for file

Additional file 3: Table S1. Details of microarray-based genomic profiling in individuals with a duplication of the *NPHP1* gene in this and previous studies. Click here for file
