# Supplementary figures and images for: Duplication of the NPHP1 gene in patients with autism spectrum disorder and normal intellectual ability: a case series
Source: Ann Gen Psychiatry. 2014 Aug 6;13:22. doi: 10.1186/s12991-014-0022-2 (PMC4131154; doi:10.1186/s12991-014-0022-2)

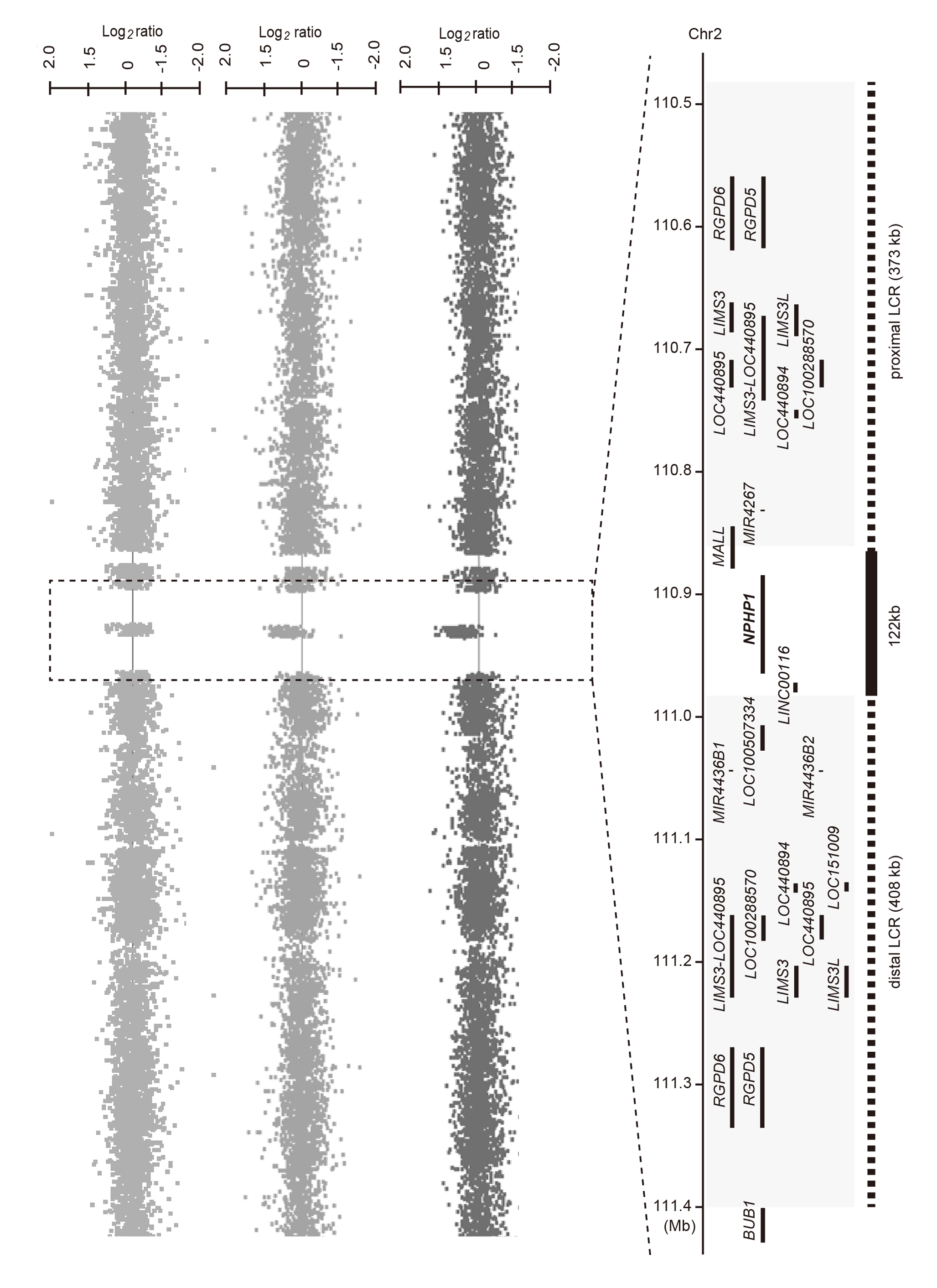

Supplement: Additional file 1: Figure S1. — De novo 2p13 duplication in two individuals with ASD. Single-nucleotide polymorphism array profiles in the 2p13 region are shown for a control patient (top), patient 1 (second row), and patient 2 (third row). The location of NPHP1, other genes, and low-copy repeats (LCRs) (gray box) are depicted based on the University of California Santa Cruz Genome Browser, build 37 (hg19) (bottom). [file s12991-014-0022-2-S1.tiff]

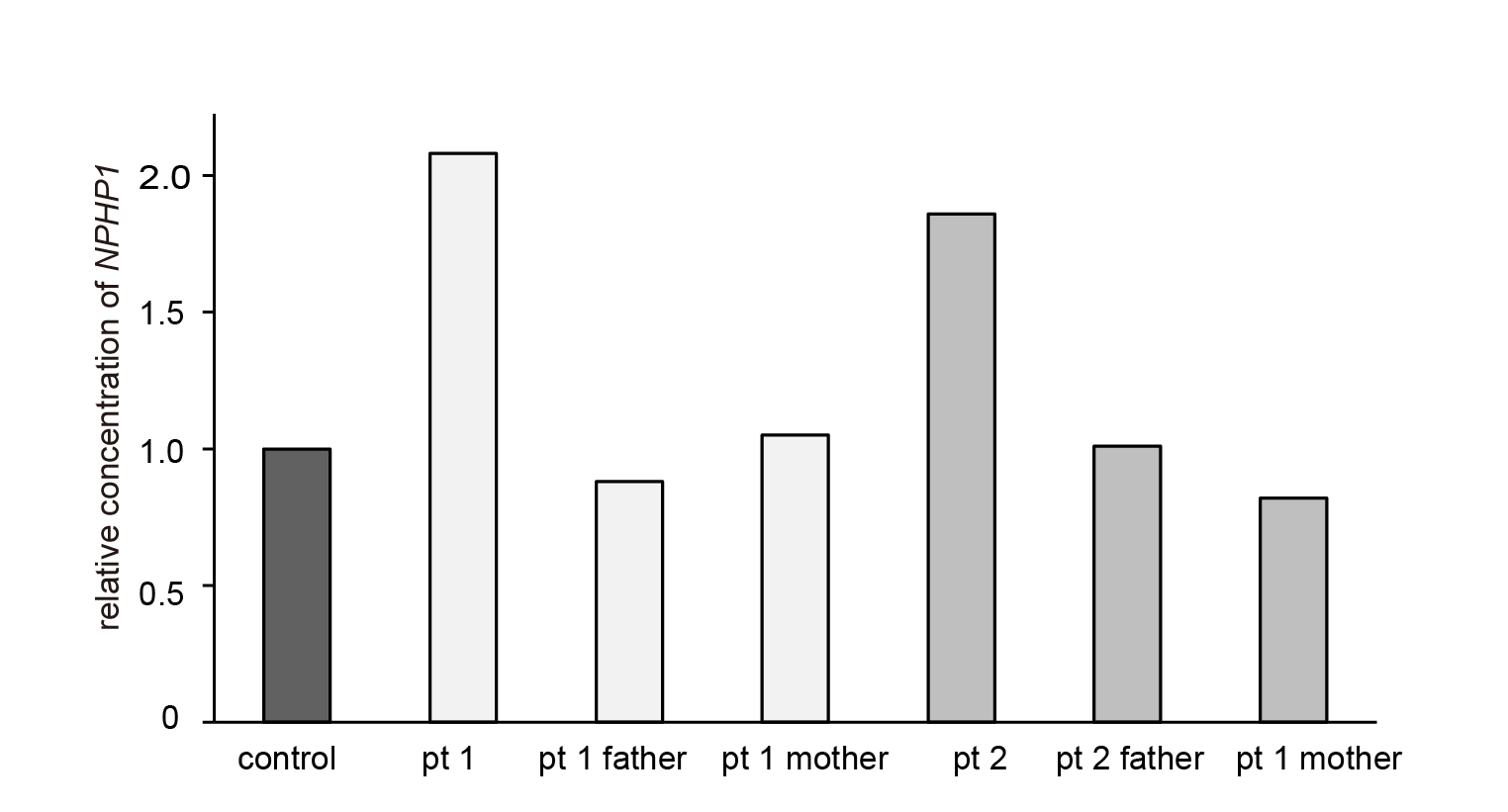

Supplement: Additional file 2: Figure S2. — Confirmation of the de novo 2p13 duplication in two individuals with ASD. Gene expression was determined by quantitative real-time PCR, and the relative concentration of NPHP1 is shown on the Y-axis. Duplication was confirmed in the two patients (Pt 1 and Pt 2) with ASD. [file s12991-014-0022-2-S2.tiff]
