# Supplementary material for: Duplication of the NPHP1 gene in patients with autism spectrum disorder and normal intellectual ability: a case series
Source: Ann Gen Psychiatry. 2014 Aug 6;13:22. doi: 10.1186/s12991-014-0022-2 (PMC4131154; doi:10.1186/s12991-014-0022-2)
Supplement: Additional file 3: Table S1. — Details of microarray-based genomic profiling in individuals with a duplication of the NPHP1 gene in this and previous studies. [file s12991-014-0022-2-S3.docx]

**Supplemental Table 1 Details of microarray-based genomic profiling in individuals with a duplication of the *NPHP1* gene in this and previous studies.**

| **Author, Year** | **Study ID** | **Cytoband** | **Size (kbp)** | **Genomic coordinates Build 37 (hg19) (minimum region of copy number changes)** | **Genes** |
| --- | --- | --- | --- | --- | --- |
| **Yasuda Y. et al., 2014** | **Patient 1** | **2q13** | **892.019** | **chr2:110496601-111388620** | **RGPD6, RGPD5, LIMS3, LIMS3 L, LIMS3-LOC440895, LOC440895, LOC100288570, LINC01123, MIR4267, MALL, MIR4436B1, MIR4436B2, NPHP1, LINC00116, LOC100507334, LINC01106** |
|  | **Patient 2** | **2q13** | **475.79** | **chr2:110504318-110980108** | **RGPD6, RGPD5, LIMS3, LIMS3 L, LIMS3-LOC440895, LOC440895, LOC100288570, LINC01123, MIR4267, MALL, MIR4436B1, MIR4436B2, NPHP1** |
| **Kaminsky EB et al., 2011** | **ISCA**  **00000020** | **2q13** | **506.415** | **chr2:110862477-111368891** | **MALL, NPHP1, LINC00116, LOC100507334, MIR4436B1, MIR4436B2, LINC01106, LINC01123, LIMS3-LOC440895, LOC100288570, LOC440895, LIMS3, LIMS3 L, RGPD6, RGPD5** |
|  | **ISCA**  **00000128** | **2q13** | **266. 371** | **chr2:110862477-111128847** | **MALL, NPHP1, LINC00116, LOC100507334, MIR4436B1, MIR4436B2** |
|  | **ISCA**  **00000949** | **2q13** | **96.651** | **chr2:110862477-110959127** | **MALL, NPHP1** |
|  | **ISCA**  **00001175** | **2q13** | **96.651** | **chr2:110862477-110959127** | **MALL, NPHP1** |
| **Pinto D et al., 2010** | **5306_4** | **2q13** | **362.711** | **chr2:110817833-111180543** | **MIR4267, MALL, MIR4436B1, MIR4436B2, NPHP1, LINC00116, LOC100507334, LINC01106, LINC01123, LIMS3-LOC440895, LOC100288570, LOC440895** |
| **Baris H et al., 2006** | **Patient 1** | **2q13** | **360.766** | **chr2:110762884-111,123,649*** | **MIR4267, MALL, MIR4436B1, MIR4436B2, NPHP1, LINC00116, LOC100507334** |

***The positions were estimated using BAC clone sequences (RP11-335A19, RP11-528G9, RP11-264O8) according to GRCh37/hg19.**
